# S645C Point Mutation Suppresses Degradation of EGFR to Promote Progression of Glioblastoma

**DOI:** 10.3389/fonc.2022.904383

**Published:** 2022-06-23

**Authors:** Wenda Huang, Ling Zou, Zhaonian Hao, Baofeng Wang, Feng Mao, Qiuhong Duan, Dongsheng Guo

**Affiliations:** ^1^ Department of Neurosurgery, Tongji Hospital, Tongji Medical College, Huazhong University of Science and Technology, Wuhan, China; ^2^ Department of Biochemistry and Molecular Biology, School of Basic Medicine, Tongji Medical College, Huazhong University of Science and Technology, Wuhan, China; ^3^ Department of Neurosurgery, Beijing TianTan Hospital, Capital Medical University, Beijing, China

**Keywords:** EGFR, S645C point mutation, glioblastoma, targeted therapy, individualized treatment, EGFR degradation

## Abstract

**Background:**

The tightly controlled activity of EGFR is important for the homeostasis of self-renewal of human tissue. Mutations in the extracellular domain of EGFR are frequent and function as a novel mechanism for oncogenic EGFR activation in GBM, and impact the response of patients to small-molecule inhibitors.

**Methods:**

We constructed glioblastoma cell lines stably expressing wild-type EGFR and the mutant of EGFR S645C. We detected cell growth *in vitro* and *in vivo.* We evaluated the anti-tumor activity and effectiveness of gefitinib and osimertinib in cells.

**Results:**

In the present study, we identified an oncogenic substituted mutation of EGFR—S645C. The mutation can promote the proliferation and colony formation of glioblastoma *in vitro* and *in vivo*. Mechanistically, the EGFR S645C mutation potentially changes the formation of hydrogen bonds within dimerized EGFR and inhibits the degradation of EGFR to prolong downstream signaling. The mutation induces resistance to gefitinib but presents an opportunity for osimertinib treatment.

**Conclusion:**

The study indicated a novel oncogenic mutation and advises on the precise treatment of individual patients with the EGFR S645C mutation.

## Introduction

Glioblastoma (GBM) is the most common and the most malignant tumor in the central nervous system (1). Despite the utility of safe surgical resection followed by radiotherapy with concomitant and adjuvant temozolomide, the prognosis of GBM patients remains dismal (2). GBM was among the first cancer types to be systematically studied by The Cancer Genome Atlas (TCGA) Research Network (3). There are plenty of abnormalities in genes and proteins that account for the pathogenesis and progression of glioma, typically involving the receptor tyrosine kinases (RTKs), p53, and retinoblastoma protein (RB) signaling pathways and therein often involving multiple RTKs such as EGFR, PDGFR, and c-MET (3, 4). Genomic profiling based on a large sample has detected that EGFR alterations occur in almost every type of tumor, especially in more than half of GBMs (5). Major genetic events of EGFR are amplification, structural variant, mutation (nucleotide substitution, deletion, and insertion), and multiple alterations, which often excessively activate EGFR and its downstream signaling cascades including RAS-RAF-MEK-ERK, PI3 kinase-AKT, PLCgamma-PKC, and STAT modules (6). Therefore, a variety of small-molecule inhibitors have been proposed and have gradually become an important part of cancer treatment, especially lung cancer. Known exon 19 deletion (del19), exon 21 (L858R) substitution mutation causing excessive activation, T790M, C797S inducing resistance to targeted oncotherapy (7, 8), and the tyrosine kinase inhibitors (TKIs) that selectively bind to EGFR function as the most important therapeutic class for cancer targeted therapy, such as gefitinib, afatinib, and osimertinib. These small-molecule inhibitors usually aim at the kinase domain (amino acids 682–955) intracellularly (9, 10), and can even be a substitute for conventional chemoradiotherapy as a first-line regimen for lung cancer. However, the TKIs did not bring similar benefits to GBM patients, and it is important to reveal the exclusive characteristics of GBM.

In contrast to lung cancer, the mutation of EGFR in GBM often takes place in the extracellular domain (ECD) instead of the kinase domain. For instance, EGFRvIII, an in-frame deletion causing truncated mutation, which lacks the extracellular ligand-binding domain due to deleting exons 2–7, can be constitutively activated to promote the growth of cancer cells in the absence of EGF and other ligands. EGFRvIII is deemed as the most frequent and characterized mutation of EGFR, which is detected in approximately 20% of GBM and only occurs in oncogenic cells (4, 11). EGFR amplification is an early event during the pathogenesis of GBM, but the occurrence of EGFRvIII shows a strong temporal and regional heterogeneity during the development of primary GBM or recurrence after surgical resection (11). The gain of EGFRvIII is deemed to be due to the amplification of extrachromosomal DNA (ecDNA) (12). The continuous activity of EGFRvIII suggests that the ECD of EGFR not only accepts the ligand EGF but also blocks the intrinsic ability of the transmembrane and cytoplasmic domains to dimerize and activate, with ligand binding releasing this block (10). The normal protein structure of EGFR plays an important role in controlling the homeostasis of the signaling cascade.

In addition to the structural variants, such as EGFRvI, vII, and vIII, the missense point mutation rate in the ECD (amino acids 25–645) is also up to 10%–15% in GBM (13). A289D/T/V missense mutation is the most frequent point mutation in the full length of EGFR in GBM, instead of L858R and other mutations in the kinase domain (14, 15). A289D/T/V mutation significantly decreases survival compared with patients harboring wild-type EGFR (median OS: 6 months vs. 15 months, 2-year survival rate: 12% vs. 22%) (4, 14). EGFR A289V in immortalized human astrocytes can maintain phosphorus EGFR after 12-h serum starvation, and introducing the mutant of EGFR into normal cells can induce malignant transformation (15). The missense mutation destroys the block effect of the ECD, which inhibits dimerization and the mutual phosphorylation of the intracellular domain (ICD) in EGFR. Moreover, R108K/G, G598V, P569L, and T263P share a similar mechanism of oncogenic receptor conversion (15–17). In addition, various point mutations occur in GBM, which consist of the heterogeneity among patients and within tumor masses. In conclusion, in contrast to lung cancer, the study of mutations in the ECD of EGFR, which could activate EGFR, is more valuable than the kinase domain in GBM. Thus far, the EGFR inhibitors being tested in clinical trials have not presented satisfactory efficacy; we believed that it is ideal to choose a personal regimen according to the characteristics of a single patient suffering from GBM (18).

In this study, we identified the S645C mutation of EGFR, the last amino acid of the ECD (19), as an oncogenic alteration. This point mutation is predominantly found in gliomas. EGFR S645C potentially changes the connection formation of hydrogen bonds during the dimerization with EGF, inducing the activation of EGFR. The alteration can suppress the degradation of EGFR and prolong the downstream cascade signaling to promote GBM cell growth. Simultaneously, EGFR S645C mediates resistance to gefitinib but presents an opportunity for the utility of osimertinib. The studies involving the mutation in the ECD of EGFR are significant for individualized precision treatment.

## Materials and Methods

### Cell Culture

U87, GL261, and HEK293T cell were purchased from the American Type Culture Collection (ATCC) and cultured in Dulbecco’s modified Eagle’s medium (Gibco) with 10% fetal bovine serum (FBS). The patient-derived adherent priGBM cell was cultured and established in our laboratory in 2016 and was cultured in DMEM with 10% FBS (20).

### Antibodies and Reagents

Anti-p-EGFR 1/2 (Y1068) (1:1,000, #3777s), anti-p-Erk (T202/Y204) (1:1,000, #4370s), anti-Erk (1:1,000, #4695s), anti-p-stat3 (Y727) (1:1,000, #9136), anti-stat3 (1:1,000, #4904), anti-p-Akt (S473) (1:500, #4060s), anti-PARP (1:1,000, #9532s), and anti-Cleaved PARP (1:1,000, #5625s) were purchased from Cell Signaling Technology. Anti-EGFR (1:1,000, 18986-1-AP), anti-Akt (1:1,000, 10176-2-AP), and anti-tubulin (1:1,000, 66031-1-Ig) were purchased from Proteintech. Anti-Flag (1:1,000, Mouse IgG, F1804), polybrene, and cycloheximide (CHX) were purchased from Sigma-Aldrich. Osimertinib and gefitinib were purchased from MedChemExpress (MCE).

### Plasmids

Phage vectors (#118692) and pCMV-C-flag vectors were purchased from Addgene and Beyotime, respectively. The plasmids of phage-EGFRwt, phage-EGFRmut, pCMV-CD533wt-flag, and pCMV-CD533mut-flag were constructed in our laboratory according to the manufacturer’s instructions (Vazyme, ClonExpress II One Step Cloning Kit, C112-01).

### MTT Assay

To estimate cell growth, cells (3×10^3^/well) were seeded in 96-well plates and cultured for 24 h, 48 h, 72 h, and 96 h. Subsequently, 3-(4,5-dimethylthiazol-2-yl)-2,5-diphenyltetrazolium bromide (MTT) was added and incubated for 4 h. Dimethyl sulfoxide (DMSO; 150 μl) was added, and absorbance value was measured at a wavelength of 490 nm when the crystal was fully dissolved. To assess osimertinib or gefitinib inhibition, cells (3×10^3^/well) were treated with inhibitors at various concentrations. Osimertinib or gefitinib cytotoxicity was measured using an MTT assay as described above.

### Anchorage-Independent Growth Assay (Soft Agar Assay)

A total of 8×10^3^ cells/well were seeded in a 6-well plate and cultured in 1 ml of 0.33% Basal Medium Eagle (BME) agar containing 10% FBS, maintained at 37°CC, 5% CO_2_ for 14–21 days. The colonies were observed under a microscope. Each group was repeated three times, averaged and multiplied by 57 (the bottom area of one well in a six-well plate is 57 times the area of one field of view of a fourfold microscope) to obtain the final quantity of the colony.

### Colony Formation Assay

Cells (2,000/well) were seeded in 6-well plates, and the cells were placed in an incubator with medium changes every 5 days. After growing to an appropriate density, the cells were washed twice with PBS and fixed with 4% paraformaldehyde for 30 min. Then, the cells were stained with 0.1% crystal violet for 30 min and washed once with PBS, and then dried and photographed.

### Edu Assay

A BeyoClick™ EdU-594 Cell Proliferation Detection Kit (Ruibo Biotech, Guangzhou, China) was used to detect cell proliferation in accordance with the manufacturer’s instructions. U87 and PriGBM cells (7,000) were seeded in 96-well plates. After 24 h, 10 μM EdU was added, and then the cells were incubated for another 4 h. This was followed by fixing with 4% paraformaldehyde for 15 min and treating with 0.5% Triton X-100 for 20 min. Then, cells were stained with DAPI for 15 min. After three washes in phosphate buffer saline (PBS), the cells were observed and photographed with an inverted fluorescence microscope (Olympus, Japan). This experiment was repeated three times.

### CHX Treatment

The cells were seeded in a 6-cm^2^ dish and treated with 80 ng/ml EGF for 15 min. Then, 50 μg/ml of CHX was added to block the new protein synthesis. Cells were harvested at a range of time points (0, 3, 6, 9, 12, and 24 h), and the levels of EGFR were analyzed by Western blot.

### Intracranial Xenograft Glioma Mouse Model

Four-week-old C57 mice were injected into the right striatum of the brain with 3×10^5^ luciferase-labeled GL261 cells overexpressing EGFRwt or EGFRmut. To monitor tumor growth in live mice, mice were intraperitoneally injected with 150 mg/kg D-luciferin and anesthetized with isoflurane on the 7th and 14th day after cell injection. The size of the tumor was monitored through the bioluminescence channel of the Spectrum Lago X imaging system. The mice were observed daily and euthanized when they showed neurological signs (a protruded skull, hunched posture, extreme lethargy, or weight loss).

### Immunohistochemical Staining

IHC staining was performed using rabbit-anti-EGFR (Proteintech, 18986-1-AP, 1:600) and rabbit-anti-Ki-67 (Servicebio, #GB13030-M-1, 1:1,000) antibodies. Expression levels of labeling were stratified and scored as previously described (21).

### Statistical Analysis

Statistical analysis was performed using GraphPad Prism 7.0 software (GraphPad, San Diego, CA). Student’s *t*-test or one-way ANOVA was used to analyze two groups or multiple groups, respectively. All data are presented as the mean ± standard deviation, and * *p* < 0.05, ** *p* < 0 01, and *** *p* < 0.001 were considered as significant.

## Results

### EGFR S645C Mutation Is Mainly Found in GBM and Potentially Changes the Connection Formation of Hydrogen Bonds in Dimerized EGFR

**Figure 1:**
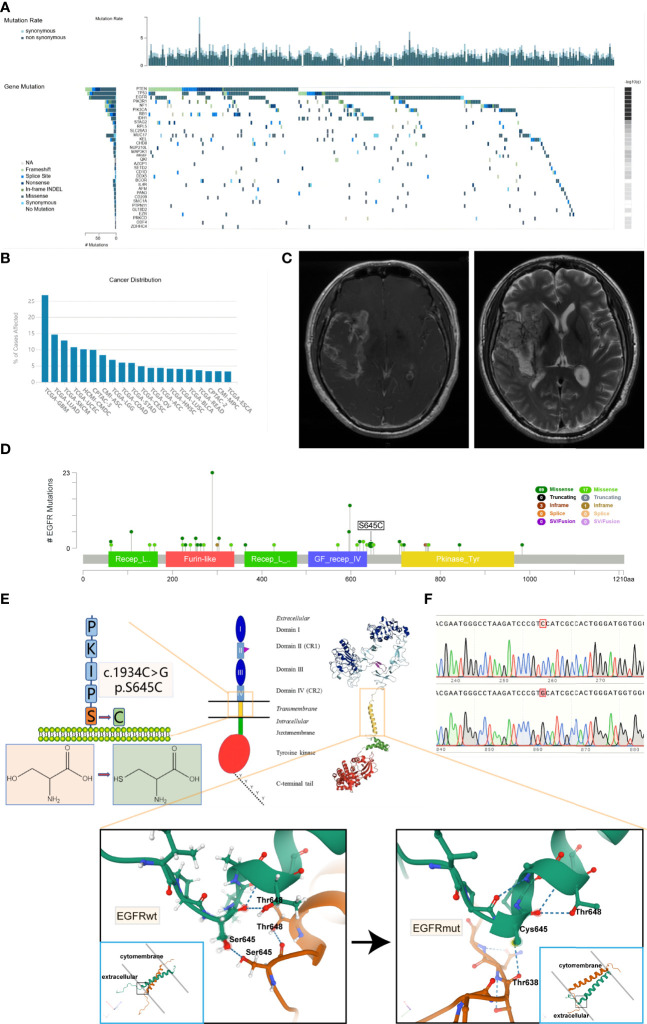
It is frequent that missense mutations in the ECD of EGFR in glioblastoma and EGFR S645C substituted mutation potentially change the formation of hydrogen bonds of dimerized EGFR. **(A)** Based on the TCGA data, the oncoplot displays the somatic landscape of the GBM cohort. Genes are ordered by their mutation frequency. The sidebar plot shows log10 transformed *Q*-values estimated by MutSigCV (TumorPortal database). **(B)** The *EGFR* gene mutation rates of multiple types of cancer (TCGA database). **(C)** The MRI results of the patient harboring EGFR S645C. **(D)** Lollipop plot displaying mutation distribution and protein domains for *EGFR* gene in GBM with the labeled recurrent hotspots (cBioportal, TCGA Firehose Legacy dataset). **(E)** A schematic representation of the structure of EGFR and the substituted mutation S645C. The 3D shape of EGFRwt is acquired from the Protein Data Bank (PDB 5LV6), and the 3D shape of EGFRmut is predicted by SWISS-MODEL using EGFRwt (PDB 5LV6) as protein structural templates. The amino acid sequence of EGFR is acquired from the UniProt database. **(F)** The peak plot results of DNC sequencing of EGFRwt and EGFRmut (c.1934C>G). ECD, extracellular domain; MRI, magnetic resonance imaging.

Based on the TCGA dataset, we have established that *PTEN*, *TP53*, and *EGFR* are the three most frequent simple somatic mutation genes in GBM (**Figure 1A**) (22). The vast majority of *EGFR* mutation is missense mutation, and the mutation rates are much higher than the fourth—*NF1*. Simultaneously, the mutation rate of *EGFR* in GBM is the highest among 33 cancer types documented in the TCGA dataset (up to 26.9%) (**Figure 1B**). Recently, we encountered a patient harboring EGFR S645C. The imaging result of MRI suggested cerebral infarction complicated by hemorrhage, but the focal area of disease is actually GBM (**Figure 1C**). The patient has a dismal prognosis of overall survival (8 months) after Stupp regimen treatment. Through online search of the TCGA, cBioportal, and COSMIC databases (23, 24), we found a total of 8 documented samples harboring the EGFR S645C mutation, and notably, there are 6 GBM samples (**Table 1**). We reasoned that EGFR S645C plays a facilitating role in the development of GBM. Somatic mutations of EGFR in GBM mainly focus on the ECD and the S645 site near domain IV of EGFR, which is indispensable for the dimerization of EGFR (**Figure 1D**) (19). Previous studies have demonstrated that domains I and III play a role in ligand binding and domains II and IV play a role in forming dimers of EGFR. To investigate how the transformation from serine to cysteine impacts the function of EGFR, the SWISS-MODEL tool was employed to predict the change in spatial conformation of dimerized EGFR (25). The results showed that the hydrogen bonds between respective S645 sites of two wild-type EGFR monomers are substituted by different bonds between C645 and the other mutational monomeric T638 (**Figure 1E**). Thus, the EGFR S645C mutation in the ECD potentially influences the activity of EGFR that should have been tightly controlled to promote the growth of GBM.

**Table 1:** All samples with EGFR S645C point mutation from the cBioPortal database.

Study of Origin	Sample ID	Cancer Type
Diffuse Glioma (GLASS Consortium, Nature 2019)	GLSS-CU-R005-TP	Glioblastoma
Glioma (MSKCC, Clin Cancer Res 2019)	P-0018572-T01-IM6	Glioblastoma Multiforme
Proteogenomic and metabolomic characterization of human glioblastoma (CPTAC, Cell 2021)	C3N-01852	Glioblastoma Multiforme
Glioblastoma Multiforme (TCGA, Firehose Legacy)	TCGA-06-2565-01	Glioblastoma Multiforme
Pediatric Preclinical Testing Consortium (CHOP, Cell Rep 2019)	NCH-CA-3	Colorectal Adenocarcinoma
Lung Adenocarcinoma (Broad, Cell 2012)	LUAD-NYU608	Lung Adenocarcinoma
Glioblastoma Multiforme (TCGA, PanCancer Atlas)	TCGA-12-0657-01	Glioblastoma Multiforme
Glioblastoma Multiforme (TCGA, PanCancer Atlas)	TCGA-12-0829-01	Glioblastoma Multiforme

### EGFR S645C Significantly Promotes the Growth of GBM Cells *In Vitro* and *In Vivo*

**Figure 2:**
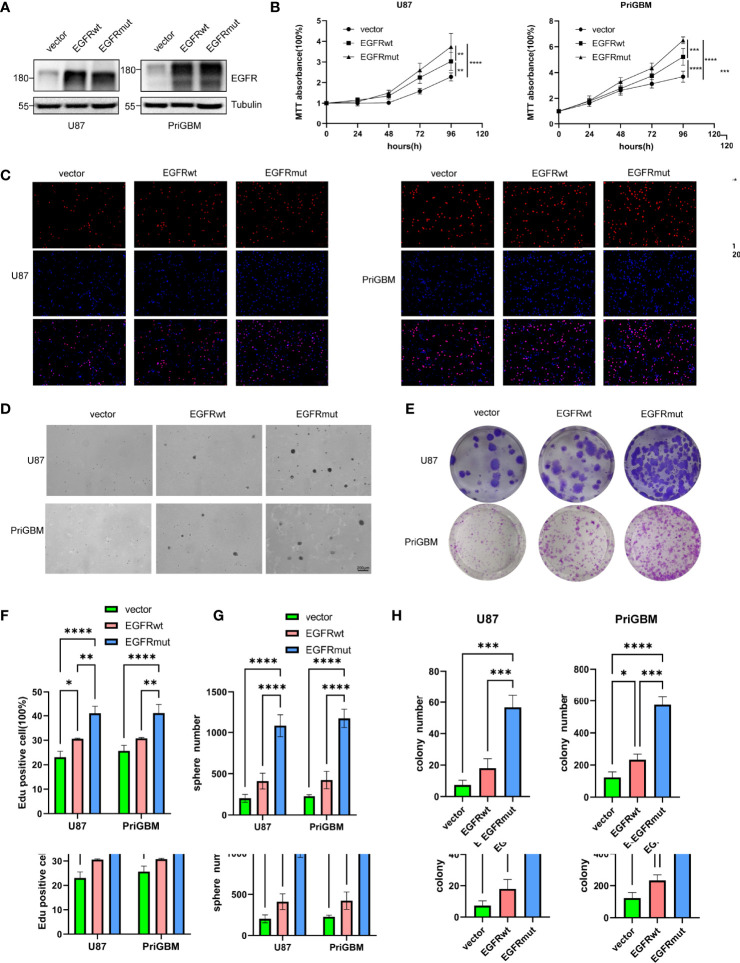
Stable expression of EGFR S645C significantly promotes the proliferation of glioma cells *in vitro*. **(A)** U87 and PriGBM cells stably expressing EGFRwt and EGFRmut were subjected to Western blotting analyses. **(B)** GBM cell viability was determined by MTT assay. **(C)** Representative imagings of BrdU assay. **(D, E)** Representative imagings of soft-agar and plate colony formation assay of GBM cells. Data are presented as means ± SD of 3 independent replicates **(F–H)**. *p < 0.05; **p < 0.01; ***p < 0.001; ****p < 0.0001.

**Figure 3:**
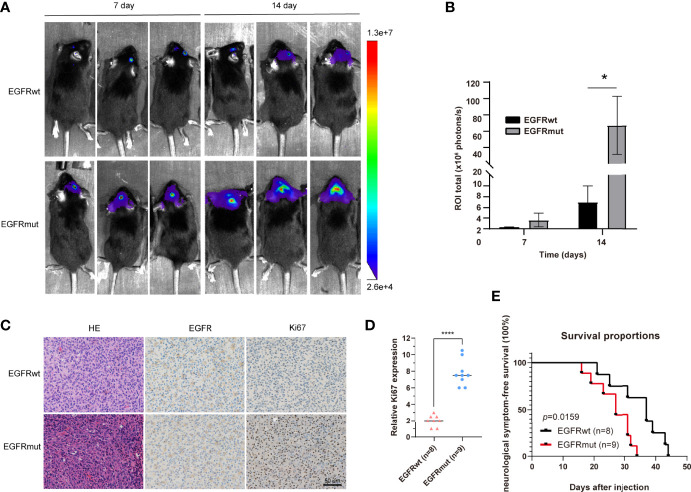
Stable expression of EGFR S645C significantly promotes the proliferation of glioma cells *in vivo*. **(A, B)** GL261 cells (3×10^5^) stably expressing EGFRwt or EGFRmut were subjected to xenograft tumor growth assay. The bioluminescence imaging and quantification of the 7th day and 14th day are presented. Data are means ± SD of 3 replicates. **(C)** H&E staining and IHC staining of EGFR, p-Erk1/2, and Ki67 in transplanted GL261 tumors. Representative photographs for each antibody and each group are shown. **(D)** Relative levels of Ki67 protein in GBM specimens of mice. **(E)** The survival curve demonstrating neurological symptom-free survival was presented (*p* = 0.0159).

Given the dismal imaging results and prognosis of the patient, we reasoned that the EGFR mutation would accelerate the development of tumor mass. To investigate the influence of the EGFR S645C mutation on the development of GBM, U87 and a primary GBM cell line—PriGBM—were employed to stably express wild-type EGFR (EGFRwt) or EGFR S645C (EGFRmut). Simultaneously, we infect the glioma cells with an empty vector to construct the corresponding blank controls (**Figures 1F**, **2A**). The MTT proliferation assays revealed a markedly higher viability of GBM cells expressing EGFRmut than EGFRwt (**Figure 2B**). Likewise, EdU proliferation assays also confirm the increase of proliferation of GBM cells expressing EGFRmut compared with EGFRwt (**Figures 2C, F**). Furthermore, expressing EGFRmut promotes colony formation as evidenced by soft agar and plate colony formation assays (**Figures 2D, E, G, H**). To confirm that EGFR S645C affects tumor growth *in vivo*, an orthotopic brain tumor model was utilized. GL261 was employed to stably express EGFRwt or EGFRmut and then was intracranially injected into C57 mice (**Supplementary Figure 1A**). After 7 days and 14 days, bioluminescent imaging demonstrated that EGFRmut promotes GL261 xenograft tumor growth *in vivo* (**Figures 3A, B**). Mice suffering from tumors with EGFRmut had shorter neurological symptom-free survival (**Figure 3E**). The results of IHC showed a higher expression of Ki67, a marker of cell proliferation activity, which is in line with the results *in vitro* (**Figures 3C, D**). Collectively, these results suggest that EGFR S645C significantly promotes the proliferation and colony formation of GBM cells compared with EGFRwt *in vitro* and *in vivo*.

Interestingly, just like EGFRvIII inducing different transcriptomes and properties of tumor cells compared with the amplification of EGFRwt (4, 11, 26, 27), EGFR S645C also potentially induces alteration of downstream genes to change the characteristics of GBM cells. We observed that there are large tumor necrotic areas in the tumor bulk of mice carrying EGFRmut rather than EGFRwt (**Supplementary Figure 1B**), consistent with the above MRI results of the patient (**Figure 1C**). The mRNA levels of several key factors associated with GBM inducing angiogenesis of tumor tissues significantly decreased, evaluated by quantitative reverse transcription PCR (qRT-PCR) (**Supplementary Figure 1C**). This is also regarded as heterogeneity within the tumor bulk of GBM caused by EGFR aberration (28).

### EGFR S645C Potentially Changes the Connecting Form of Dimerized EGFR and Sensitizes the Receptor to EGF

**Figure 4:**
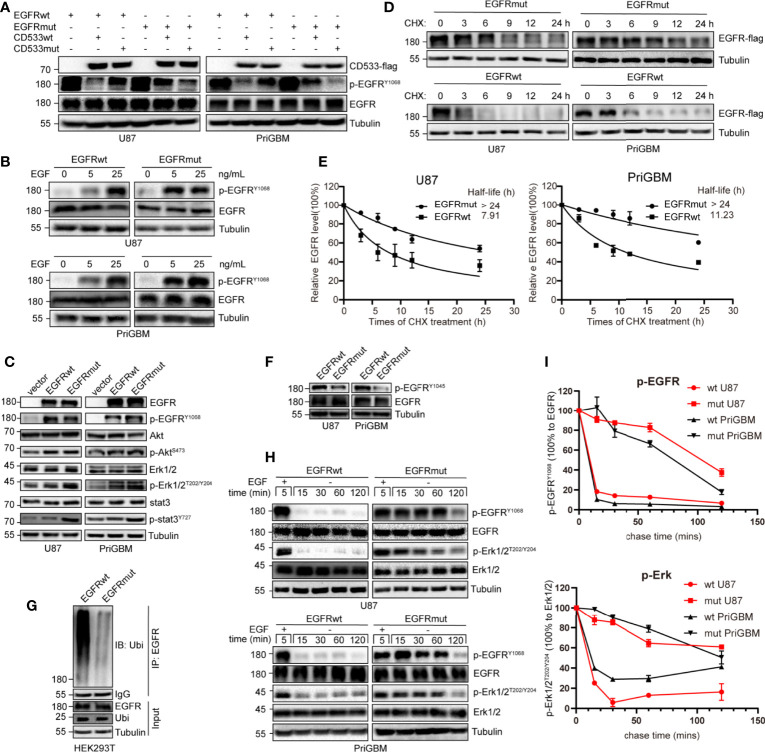
S645C mutation suppresses the degradation of EGFR and prolongs the downstream signaling by inhibiting the ubiquitination of EGFR. **(A)** The EGFR-CD533wt and CD533mut were transiently expressed in U87 and PriGBM, which stably express EGFRwt and EGFRmut. After 48 h, the cells were harvested and were subjected to Western blotting analyses. **(B)** U87 and PriGBM cells were serum-starved for 12 h and then treated with different concentrations of EGF for 5 min. Cell lysates were subjected to Western blotting analysis of active EGFR. **(C)** Western blotting analysis of active EGFR and downstream signaling in U87 and PriGBM, which stably express blank vector, EGFRwt-flag, and EGFRmut-flag. **(D, E)** U87 and PriGBM cells were serum-starved for 12 h and then treated with 80 ng/ml EGF for 15 min. Sequentially, cells were treated with 50 μg/ml cycloheximide (CHX) for an indicated time interval. Cell lysates were subjected to Western blot analyses. Three independent experiments were performed. The EGFR protein levels were quantified and a plot representing protein half-life was presented as means ± SD. **(F)** Western blotting analysis of p-EGFR^Y1045^. **(G)** HEK293T cells were co-transfected with Ubiquitin and EGFRwt-flag or EGFRmut-flag plasmids. After 48 h, the cell lysates were subjected to IP-Western analysis. **(H, I)** After being serum-starved for 12 h, U87 and PriGBM cells were treated with 10 ng/ml EGF for 5 min. Then, cells were washed and further incubated for the indicated times (Chase Time) without EGF. Active EGFR and MEK1/2 were detected in cell lysates by Western blotting. The mean amounts of active EGFR and MEK1/2 normalized to total EGFR and MEK1/2, respectively, from three experiments plotted against chase time are presented.

As per the SWISS-MODEL prediction, EGFR S645C perhaps changes the hydrogen bonds surrounding the 645th site, the last amino acid of the ECD. To further confirm the alteration, we utilized a dominant-negative mutant of EGFR—EGFR-CD533—to examine the impact of the EGFR S645C mutation on the dimerization of activated EGFR (29). The mutant of EGFR lacks the COOH-terminal 533 amino acids (the entire cytoplasmic domain) and acts as a potent inhibitor of EGFR, due to its inability to mutually phosphorylate after the dimerization of EGFR (**Supplementary Figure 1D**) (30, 31). We induced the GBM cells stably expressing EGFRwt and EGFRmut to overexpress EGFR-CD533wt or EGFR-CD533 S645C (CD533mut). We found that CD533wt and CD533mut markedly suppress the phosphorylation of EGFRwt and EGFRmut, respectively, but after exchanging CD533wt and CD533mut, the effect of CD533 that inhibits the activation of EGFR is significantly reduced (**Figure 4A**). The results suggested that two EGFRwt monomers or two EGFRmut monomers are capable of effectively binding to each other after EGF stimulation, but one EGFRwt monomer and another EGFRmut monomer are disabled. Thus, we inferred that EGFR S645C changes the connecting form of dimerized EGFR. Simultaneously, EGFRmut is more sensitive to low-concentration EGF, suggesting that EGFRmut possesses higher activation efficiency (**Figure 4B**). It is different from EGFR A289V, which is basally phosphorylated in the absence of EGF (15). Furthermore, we examined the activities of downstream signals of EGFR, such as PI3K/Akt, MEK/Erk, and STAT3. Compared with EGFRwt, stably expressing EGFRmut induces an increase in pSTAT3, evidenced by Western blotting (**Figure 4C**). Together, these results indicate that EGFR S645C is an activation mutation and an oncogenetic mutation.

### S645C Mutation Inhibits the Degradation of EGFR and Prolongs the Activation of Downstream Pathways

S645C mutation is capable of influencing the activity of EGFR as per the above results; however, the phosphorylation level of EGFR Y1068 and some downstream signals, such as PI3K/Akt and MEK/Erk, does not obviously increase (**Figure 4C**). Therefore, we speculated that there are other mechanisms that account for the promising efficacy of EGFR S645C. The activity of RTKs including EGFR is not only controlled by the extent of phosphorylation but also regulated by the duration of activated status (32). Firstly, we employed CHX treatment to detect the stability and quantify the half-life of EGFRwt and EGFRmut. The results indicated a remarkable increase in the half-life of up to more than 24 h, mediated by S645C mutation in the presence of EGF (**Figures 4D, E**). Previous studies have shown that after ligand binding to and stimulating EGFR, which resides on the plasma membrane, EGFR would be endocytosed into plasma. Subsequently, a part of EGFR within early endosomes recycles back to the plasma membrane, and the residue is sorted for degradation (33, 34). Ubiquitination of EGFR induced by Cbl (an E3 ubiquitin ligase with the RING domain) is an important means to mediate endocytosis, sorting, and degradation (32, 35, 36). Cbl recognizes the phosphorylated Y1045 site of EGFR and recruits ubiquitin to conjugate with EGFR, and the process is critically important in determining EGFR stability (37). We found that EGFR S645C significantly reduces the phosphorylation level of the Y1045 site (**Figure 4F**). Furthermore, the ubiquitination level of EGFRmut after EGF stimulation is significantly decreased compared with EGFRwt (**Figure 4G**). In summary, the results demonstrate that the S645C point mutation increases the stability of EGFR. In addition, stimulating with EGF, followed by withdrawing and washing, results in the slower decay of phosphorylated EGFRmut and the slower dephosphorylation of downstream p-Erk1/2 than EGFRwt (**Figures 4H, I**). The results indicate that increasing the stability of EGFR is vital to prolong the signal cascade (33, 38, 39). Collectively, these results indicate that EGFR S645C increases the stability of EGFR and prolongs the downstream signal cascade to function as an oncogenic mutation.

### EGFR S645C Mutation Induces Resistance to Gefitinib

**Figure 5:**
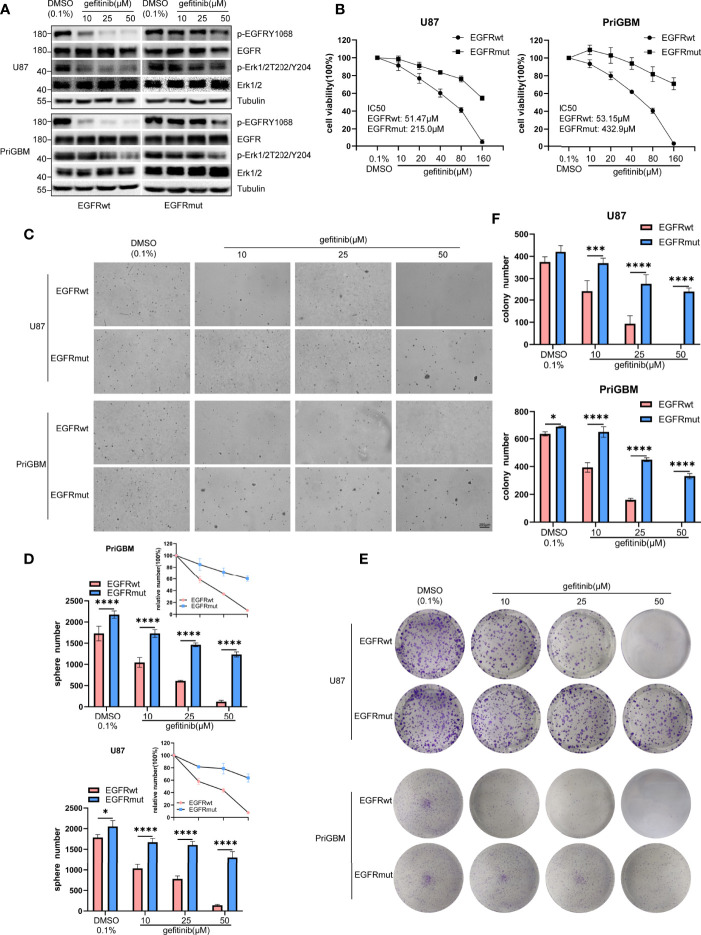
EGFR S645C induces resistance to gefitinib. U87 and PriGBM cells were treated with different concentrations of gefitinib for 48 h. **(A)** The cell lysates were subjected to Western blotting. **(B)** Cell viability was analyzed by MTT assay. **(C, D)** Representative imagings and quantification of soft-agar colony formation assay of GBM cells. **(E, F)** Representative imagings and quantification of plate colony formation assay of GBM cells. Data are presented as means ± SD of 3 independent replicates. *p < 0.05; ***p < 0.001; ****p < 0.0001.

Previous studies suggested that the point mutation of EGFR is an important way for tumor cells to develop resistance to several TKIs. T790M and C797S are among the most characteristic mutations (40, 41). Missense mutation occurring in the ECD is also a considerable approach to induce resistance (42, 43). In 2016, Berger et al. utilized high-throughput phenotyping analysis and demonstrated that rare mutations could be functionally significant, and the S645C mutation induces resistance of lung cancer cells to erlotinib (44). Gefitinib and erlotinib as first-generation small-molecule inhibitors reversibly target EGFR. However, the first-generation TKIs exhibit great potency to bind wild-type EGFR, thus causing numerous side effects such as severe diarrhea and dermatitis (45). Tumor cells often possess EGFR amplification or overactive mutants of EGFR, such as del 19 and L858R, which leads to greater sensitivity to gefitinib and erlotinib. They are gradually accepted as first-line therapy in lung cancer and are simultaneously tested in multiple types of tumor, including GBM (46, 47). In this study, we examined the sensitivity of GBM cells harboring EGFR S645C to gefitinib. Firstly, we found that gefitinib can significantly inhibit phosphorylation of EGFRwt but not EGFRmut and downstream signaling substrate (p-Erk1/2) in U87 and PriGBM determined by immunoblotting (**Figure 5A**). Consistently, the IC_50_ values of gefitinib treating U87 and PriGBM harboring EGFRwt are 51.47 μM and 53.15 μM, respectively. However, the value of IC_50_ for EGFRmut is up to 215.0 μM and 432.9 μM, evidenced by MTT cell viability assays (**Figure 5B**). Furthermore, gefitinib can significantly inhibit U87 and PriGBM expressing EGFRwt clonogenicity in soft agar and on plates instead of EGFRmut, which is in line with the above results (**Figures 5C–F**). Together, these results indicated that EGFR S645C induces resistance to a first-generation EGFR TKI—gefitinib.

### EGFR S645C Is Sensitive to Osimertinib and Presents Opportunities for Targeted Therapy

**Figure 6:**
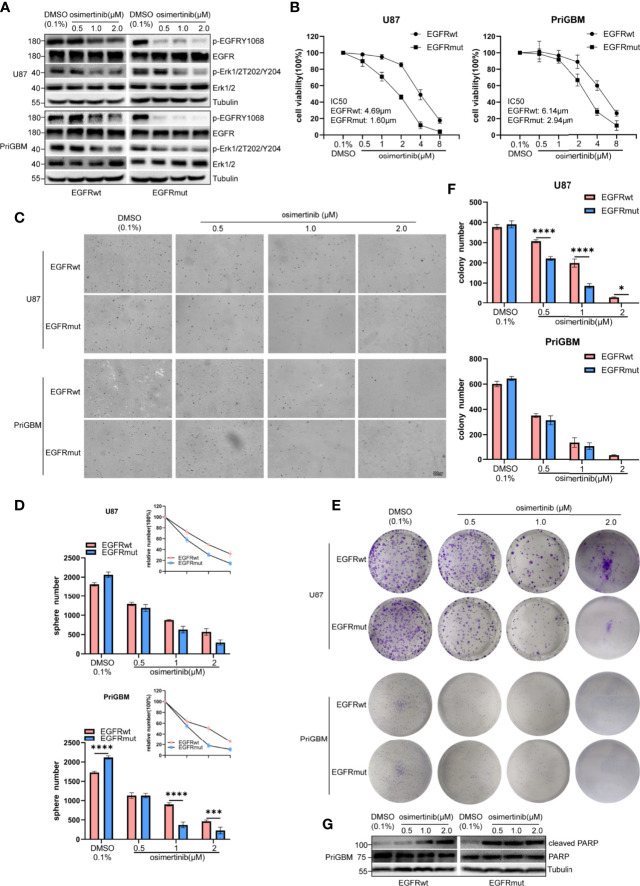
EGFR S645C presents an opportunity for Osimertinib therapy. U87 and PriGBM cells were treated with different concentrations of Osimertinib for 48 h. **(A)** The cell lysates were subjected to Western blotting. **(B)** Cell viability was analyzed by MTT assay. **(C, D)** Representative imagings and quantification of soft-agar colony formation assay of GBM cells. **(E, F)** Representative imagings and quantification of plate colony formation assay of GBM cells. Data are presented as means ± SD of 3 independent replicates. *p < 0.05; ***p < 0.001; ****p < 0.0001. **(G)** Western blotting analysis of cleaved PARP [poly(ADP-ribose) polymerase] and total PARP after being treated with different concentrations of Osimertinib for 48 h.

To resolve the acquired resistance to first-generation TKIs, many new components have been proposed to irreversibly bind mutants of EGFR. AZD9291, also known as osimertinib, is the most characteristic one. EGFR S645C continuously activates downstream signaling including Erk1/2 as mentioned previously, and MEK inhibitor can partly counter the resistance induced by EGFR S645C in lung cancer (44). Liu et al. in 2019 revealed that osimertinib can overcome the resistance of GBM to first- and second-generation TKIs by continuously inhibiting Erk signaling (48). In this study, we examined the potency of osimertinib targeting EGFR S645C. Firstly, we detected the level of p-EGFR and p-Erk1/2 in the presence of a gradient concentration of osimertinib, and found that osimertinib showed a more effective ability to suppress EGFRmut than EGFRwt, despite both showing greater effectivity than gefitinib (**Figure 6A**). The result is consistent with characteristics of the third-generation inhibitor osimertinib, which is selectively designed for overactivated mutants of EGFR and the lower binding affinity to wild-type EGFR (49). Then, we employed MTT cell viability assays to test IC_50_ (U87 EGFRwt: 4.69 μM, EGFRmut: 1.60 μM; PriGBM EGFRwt: 6.14 μM, EGFRmut: 2.94 μM, **Figure 6B**). The clonogenicity evidenced by soft agar and plate clone formation assays is in line with the results (**Figures 6C–F**). Simultaneously, osimertinib is more capable of inducing apoptosis in targeting EGFRmut rather than EGFRwt, evidenced by higher cleaved PARP [poly(ADP-ribose) polymerase] (**Figures 6G**). In conclusion, osimertinib plays a potent role in inhibiting EGFR S645C to tackle the resistance caused by the missense mutation in the ECD of EGFR.

## Discussion

GBM is the most refractory tumor causing many cancer-related deaths worldwide. Scientists and clinicians have put considerable effort to search for effective therapies for GBM in the past decades. When it comes to targeted therapy in GBM, the main approaches are anti-angiogenesis and targeting RTKs and downstream signaling proteins. Thus far, bevacizumab is the only targeted drug approved by the FDA for GBM. In GBM, truncated mutation and substitute mutation occurring in the ECD of EGFR are the main mutants (14). These mutants often show higher activity than wild-type EGFR, thus playing a huge role in the proliferation and invasion of GBM and inducing GBM cells to rely on the strong signals to promote growth (11, 14). Hence, it is ideal to take advantage of the difference between normal and tumor tissue in targeted therapy.

In this study, we firstly present a clinical patient suffering from GBM with the EGFR S645C mutation with terrible imaging results and a dismal prognosis. We employed the SWISS-MODEL and demonstrate that EGFR S645C potentially changes the hydrogen bonds to impact the dimerization and activity of EGFR. The EGFR S645C mutation inhibits the degradation of EGFR and continuously activates downstream signaling to promote the proliferation of GBM cells *in vitro* and *in vivo*. Therefore, EGFR S645C is inferred as an oncogenic mutation. In addition, we demonstrated that EGFR S645C is resistant to the first-generation EGFR inhibitor gefitinib, but the third-generation inhibitor osimertinib showed enough efficacy to suppress the proliferation induced by EGFRmut.

Up to now, there are a number of clinical trials examining various small-molecule inhibitors to treat newly diagnosed and progressing GBM. However, due to the disappointing outcomes of the trials, there is no effective regimen; thus, the best option of GBM patients is to enter clinical trials. On the one hand, GBM and the tumor-associated microenvironment are exclusively compared with other types of solid tumors, such as the occurrence of the blood–brain barrier and the heterogeneity within the tumor mass. On the other hand, the various small-molecule inhibitors are mainly designed for lung cancer instead of GBM. In addition to small-molecule inhibitors, monoclonal antibodies (mAbs) are another important part of targeted therapy, which mainly target extracellular proteins or ECD of transmembrane proteins because of the large molecular weight compared with small-molecule inhibitors. Moreover, immunological therapies, including CAR-T cell and DC cell tumor vaccines, gradually capture the attention of scientists and clinicians. EGFRvIII is often regarded as a dominant target for immunological therapies due to the exposure of an extracellular, unique, and targetable epitope (50, 51). The substituted mutations in the ECD of EGFR similarly present opportunities for monoclonal antibodies. Binder et al. indicated that mAb806 binds EGFR A298V significantly better than wild-type EGFR (14).

Substituted mutations in the ECD of EGFR also function as an inescapable means to induce resistance to EGFR inhibitors. Tumor cells respond to the stress of therapy and evolve to a resistant lineage. In lung cancer, developing mutations in the kinase domain is the major approach. Mutation in the ECD is also a method to induce resistance in tumor cells (42, 44), and as for GBM, mutations in the ECD perhaps play a more important role.

## Data Availability Statement

The datasets presented in this study can be found in online repositories. The names of the repository/repositories and accession number(s) can be found in the article/**Supplementary Material**.

## Ethics Statement

The animal study was reviewed and approved by the Ethical Committee of Tongji Hospital, Tongji Medical College, Huazhong University of Science and Technology.

## Author Contributions

WH, LZ, QD, and DG designed the study. ZH collected the information of the patient. WH and LZ performed the experiments. WH, LZ, and ZH analyzed data and drafted the manuscript. BW and FM revised the manuscript. All authors contributed to the article and approved the submitted version.

## Funding

This project was supported by the National Natural Science Foundation of China (grant no. 81874086) and by the Natural Science Foundation of Hubei Province of China (grant no. 2018CFB579).

## Conflict of Interest

The authors declare that the research was conducted in the absence of any commercial or financial relationships that could be construed as a potential conflict of interest.

## Publisher’s Note

All claims expressed in this article are solely those of the authors and do not necessarily represent those of their affiliated organizations, or those of the publisher, the editors and the reviewers. Any product that may be evaluated in this article, or claim that may be made by its manufacturer, is not guaranteed or endorsed by the publisher.

## Supplementary Material

Supplementary Figure 1
**(A)** GL261 cells were transfected with vector or stably expressed EGFRwt and EGFRmut. The cell lysates were subjected to western blotting analysis. **(B)** Representative H&E stained sections of GL261 tumors. **(C)** qRT-PCR analysis targeting angiogenic factors in PriGBM cells transduced with vector, EGFRwt, or EGFRmut. **(D)** A schematic representation of the structure of EGFR dimer consists of two EGFR monomers or an EGFR monomer and EGFR-CD533.

Supplementary Figure 2 The phosphorylation levels of Akt were detected by western blotting. **(A)** After serum-starved for 12 hours, U87 and PriGBM cells were treated with 10 ng/mL EGF for 5 minutes. And then cells were washed and further incubated for indicated times (Chase Time) without EGF. The phosphorylation levels of Akt were detected in cell lysates by western blotting. **(B)** U87 and PriGBM cells which stably express EGFRwt or EGFRmut were treated with different concentrations of gefitinib for 48 hours. The phosphorylation levels of Akt were detected by western blotting. **(C)** U87 and PriGBM cells which stably express EGFRwt or EGFRmut were treated with different concentrations of Osimertinib for 48 hours. The phosphorylation levels of Akt were detected by western blotting.
