# Supplementary figures and images for: S645C Point Mutation Suppresses Degradation of EGFR to Promote Progression of Glioblastoma
Source: Front Oncol. 2022 Jun 23;12:904383. doi: 10.3389/fonc.2022.904383 (PMC9259983; doi:10.3389/fonc.2022.904383)

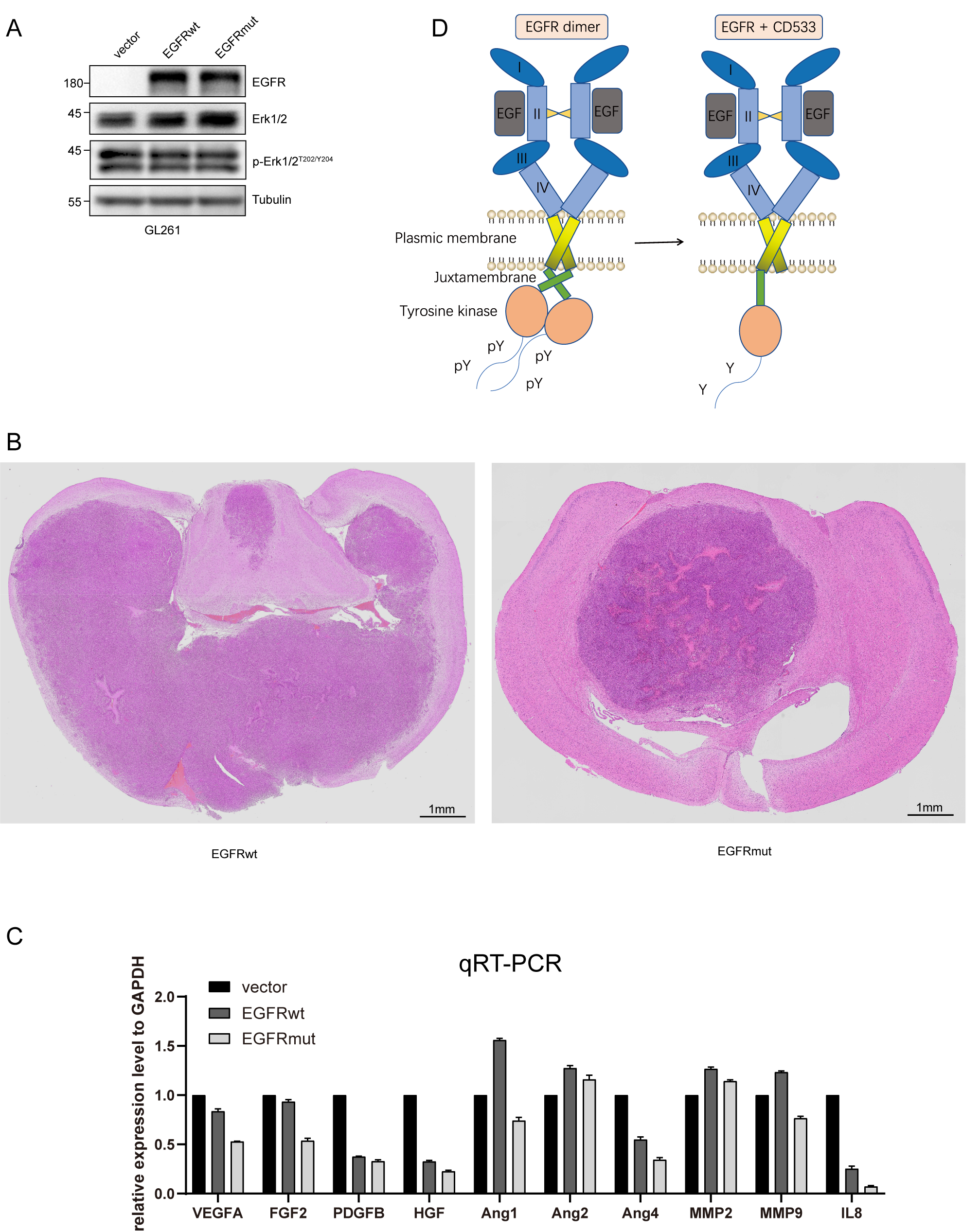

Supplement: Supplementary Figure 1 — (A) GL261 cells were transfected with vector or stably expressed EGFRwt and EGFRmut. The cell lysates were subjected to western blotting analysis. (B) Representative H&E stained sections of GL261 tumors. (C) qRT-PCR analysis targeting angiogenic factors in PriGBM cells transduced with vector, EGFRwt, or EGFRmut. (D) A schematic representation of the structure of EGFR dimer consists of two EGFR monomers or an EGFR monomer and EGFR-CD533. [file Image_1.tif]

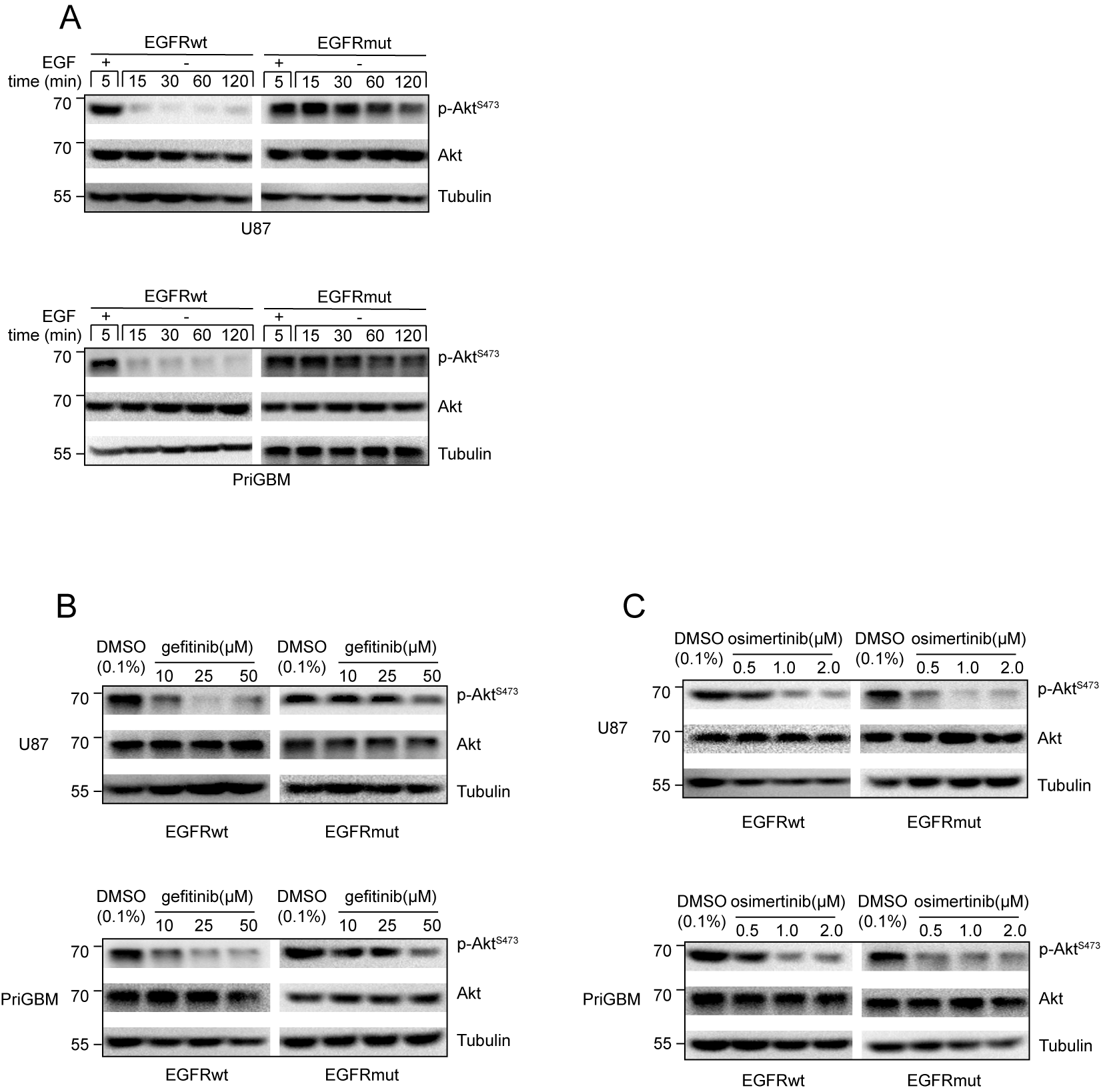

Supplement: Supplementary Figure 2 — The phosphorylation levels of Akt were detected by western blotting. (A) After serum-starved for 12 hours, U87 and PriGBM cells were treated with 10 ng/mL EGF for 5 minutes. And then cells were washed and further incubated for indicated times (Chase Time) without EGF. The phosphorylation levels of Akt were detected in cell lysates by western blotting. (B) U87 and PriGBM cells which stably express EGFRwt or EGFRmut were treated with different concentrations of gefitinib for 48 hours. The phosphorylation levels of Akt were detected by western blotting. (C) U87 and PriGBM cells which stably express EGFRwt or EGFRmut were treated with different concentrations of Osimertinib for 48 hours. The phosphorylation levels of Akt were detected by western blotting. [file Image_2.tif]
